# Early Detection of T cell Transfer-induced Autoimmune Colitis by In Vivo Imaging System

**DOI:** 10.1038/srep35635

**Published:** 2016-10-20

**Authors:** Yu-Ling Chen, Yi-Ting Chen, Cheng-Feng Lo, Ching-I Hsieh, Shang-Yi Chiu, Chang-Yen Wu, Yu-Shan Yeh, Shu-Hsuan Hung, Po-Hao Cheng, Yu-Hsuan Su, Si-Tse Jiang, Hsian-Jean Chin, Yu-Chia Su

**Affiliations:** 1 National Laboratory Animal Center, National Applied Research Laboratories, Taipei, Taiwan; 2 Institute of Molecular Biology, Academia Sinica, Taipei, Taiwan

## Abstract

Inflammatory bowel disease is a chronic and progressive inflammatory intestinal disease that includes two major types, namely ulcerative colitis and Crohn’s disease (CD). CD is characterized by intestinal epithelial hyperplasia and inflammatory cell infiltration. Transfer of CD25^−^CD45RB^hi^CD4^+^ (naïve) T cells into immunodeficiency mice induces autoimmune colitis with pathological lesions similar to CD and loss of body weight 4 weeks after cell transfer. However, weight loss neither has sufficient sensitivity nor totally matches the pathological findings of CD. To establish an early and sensitive indicator of autoimmune colitis model, the transferred T cell-induced colitis mouse model was modified by transferring luciferase-expressing donor T cells and determining the colitis by *in vivo* imaging system (IVIS). Colitis was detected with IVIS 7–10 days before the onset of body weight loss and diarrhea. IVIS was also applied in the dexamethasone treatment trial, and was a more sensitive indicator than body weight changes. All IVIS signals were parallel to the pathological abnormalities of the gut and immunological analysis results. In summary, IVIS provides both sensitive and objective means to monitor the disease course of transferred T cell-induced CD and fulfills the 3Rs principle of humane care of laboratory animals.

Inflammatory bowel disease (IBD), a high-incidence chronic intestinal inflammatory disease, affects approximately 1.4 million individuals in the United States and 2.2 million in Europe^1^. The clinical signs of IBD are body weight loss, severe diarrhea, rectal bleeding, abdominal pain, and fever. IBD is of two major types, ulcerative colitis and Crohn’s disease (CD), which are defined by the locations and pathological findings. Ulcerative colitis is restricted to the colon and cecum, with superficial mucosal and submucosal ulcers. CD affects the entire gastrointestinal tract, especially the terminal ileum and colon, with transmural discontinuous granulomatous inflammation and hyperplasia of the intestinal epithelium^2^,^3^,^4^,^5^,^6^.

The etiology of IBD is still not clear. In general, the major cause is dysregulation of immune responses induced by environmental or genetic factors. Thus, many genetically modified mouse models, chemical-induced models, and the T cell-transfer model have been established for IBD study^4^. All these animal models indicate that the T cell-mediated autoimmune response plays an important role. In these IBD animal models, transfer of naïve (CD4^+^ CD45RB^hi^) T cells into congenic immunodeficiency mice (T cell transfer colitis model), which is known as a good CD model, is one of the most common models. The advantage of the T cell transfer colitis model is the closer synchronization of the onset and severity of disease as compared to other models. Many publications share very detailed experimental procedures for establishing a T cell transfer colitis model^7^,^8^,^9^. The guidelines for successfully establishing a T cell transfer colitis model include the purity and viability of donor naïve T cells and a high-level SPF barrier environment without *Helicobacter spp., Pneumocystis carinii,* and mouse hepatitis virus contaminations^7^. Critical measurements of this model are changes in body weight (BW), diarrhea onset, and pathological observations at the endpoint of the experiment. However, loss of BW and diarrhea onset are usually found 3–5 weeks after adoptive transfer, and the host mice can survive only 1–2 weeks after diarrhea onset. In addition, some host mice may not exhibit clinical symptoms but still develop classical pathological lesions. This raises an important question: Is there any other observation for evaluation of the progress of autoimmune colitis in the T cell transfer colitis model?

We modified the traditional T cell transfer colitis model by using luciferase-expressing (Luc-expressing) naïve T cells as donor naïve T cells and determined the bioluminescence imaging (BLI) of host mice with an *in vivo* imaging system (IVIS). The results showed that BLI analysis can detect onsets of autoimmune colitis in host mice transferred with Luc-expressing naïve T cells earlier than changes in BW in the traditional T cell transfer colitis model. The BLI results also show good correlation with the pathological scoring of colitis. This study provides an objective and measurable basis for judging the starting point of therapeutic trials and increases the treatment window by 1–2 weeks relative to that of the traditional model.

## Results

### Early detection of abdominal inflammation by BLI analysis

**Figure 1:**
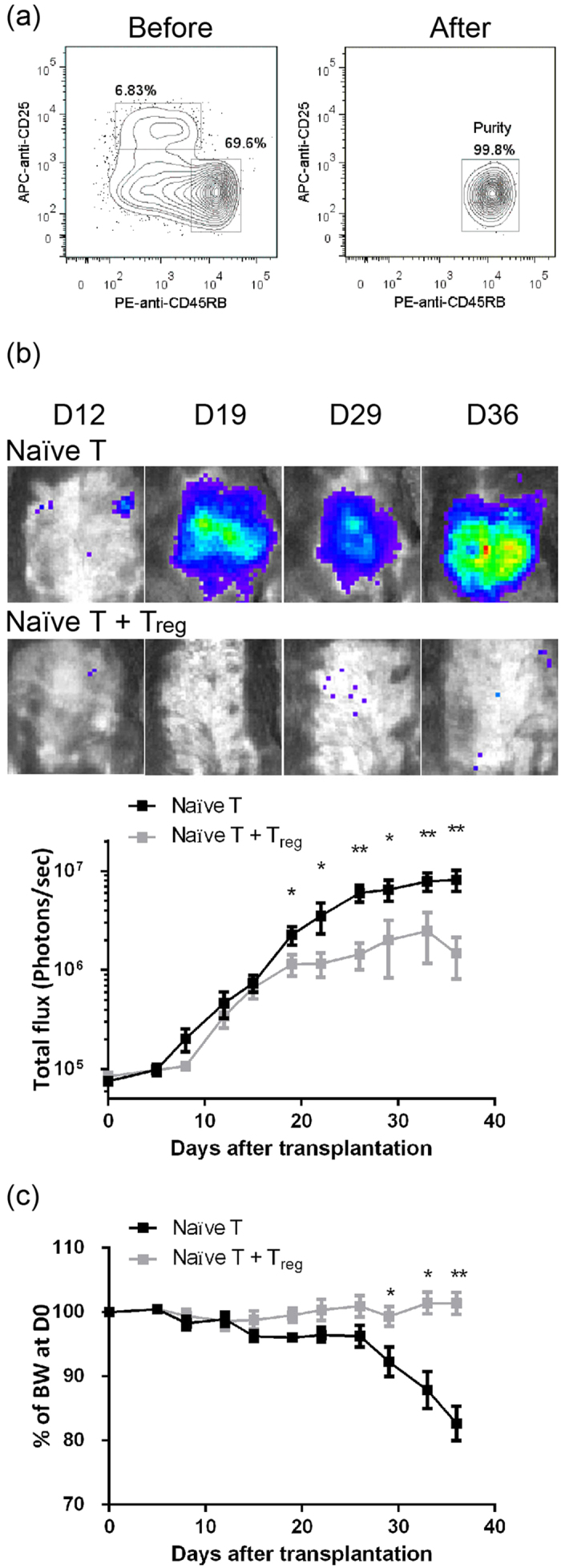
Early detection of autoimmune colitis by BLI analysis. (**a**) Percentages of CD4^+^ CD25^−^CD45RB^hi^ naïve T cells from Luc Tg. spleens before and after sorting are shown. (**b**) Continued BLI analysis of Luc-expressing, naïve T cell-transferred Rag1-ko mice. Rag1-ko host mice that received Luc-expressing naïve T cells or naïve T cells + CD4^+^ CD25^+^ CD45RB^lo^ T_reg_ cells were analyzed with IVIS twice a week. Abdominal BLIs were significantly detected at D12-D15 PAT and thereafter. Total influx at each time point after adoptive transfer is shown. (**c**) Changes of BW of Rag1-ko host mice after transfer of Luc-expressing naïve T cells. BW shown at each time point is normalized by the BW at D0. Data from four repeated experiments are combined (naïve T, n = 14; naïve T + T_reg_, n = 14) and represented by Means ± SEM. (*p < 0.05, **p < 0.01).

After Luc-expressing naïve T cells were adoptively transferred into Rag1-ko host mice (The purity of donor naïve T cells was >95%, Fig. 1a), abdominal BLIs of the host mice were analyzed twice a week (Fig. 1b). BLI of the host mice received Luc-expressing naïve T cells or naïve T cells + Regulatory T (T_reg_) cells increased after transfer and reached ~4 × 10^5^ photons/sec at D15 post adoptive transfer (PAT). BLIs of host mice that received Luc-expressing naïve T cells continuously increased with the course of time to around 8 × 10^6^ photons/sec at endpoint. On the other hand, weak BLIs of the host mice that received naïve T + T_reg_ cells reached the top point around only 1–2 × 10^6^ photons/sec at D19 PAT. Compared with the mice that received naïve T + T_reg_ cells, the BLIs of the naïve T group were significantly higher from D19 PAT and thereafter (D19, *p* = 0.03; D22, *p* = 0.009; D26, *p* = 0.004; D29, *p* = 0.006; D33, *p* = 0.009; D36, *p* = 0.014).

The most common indicator for the autoimmune colitis mouse model in life is BW loss. The BWs of both the mice that received naïve T and those that received naïve T + T_reg_ cells showed no changes until D25 PAT (Fig. 1c). While the naïve T + T_reg_ group maintained BW, the BW of mice that received naïve T cells decreased by ~8% at D29 PAT and then dropped by 17% within one week. The BW of mice that received naïve T cells decreased significantly more than the BW of the naïve T + T_reg_ group at D33 and D36 PAT (D33, *p* = 0.019; D36, *p* = 0.003). Therefore, BLI analysis is much more sensitive and can detect the onset of abdominal inflammation 10 days earlier than BW loss.

### Luc-expressing T cells induced severe colitis and moderate enteritis

**Figure 2:**
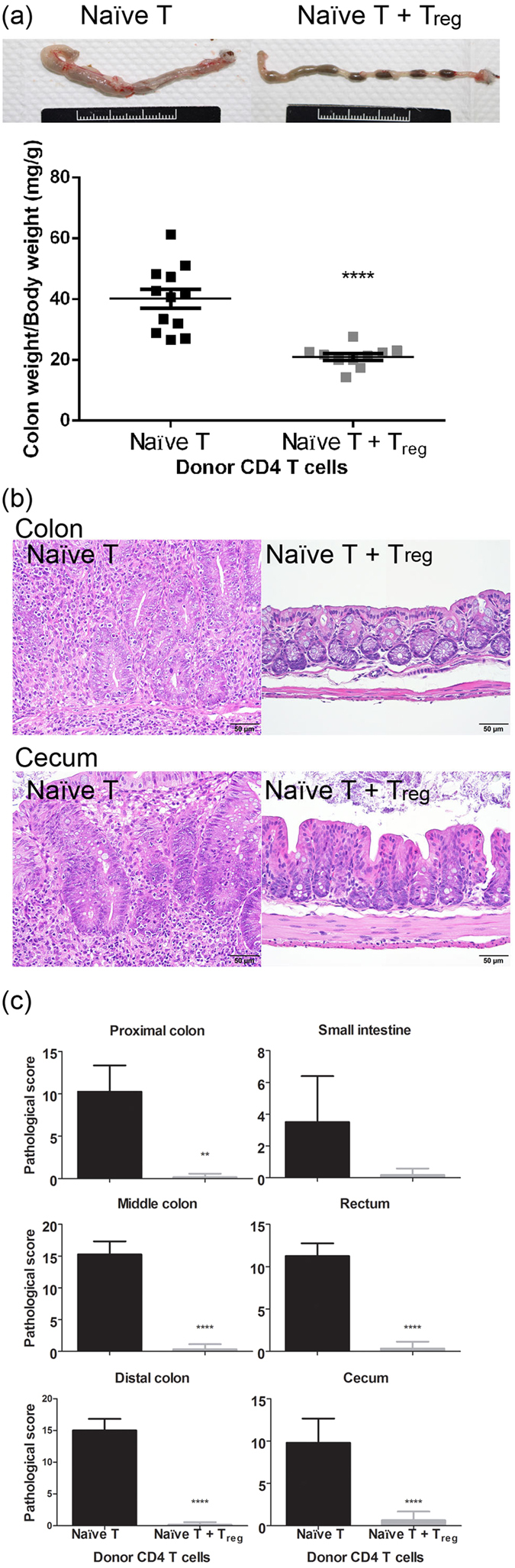
Luc-expressing T cells induced severe colitis and moderate enteritis. (**a**) Enlargement of colons from host mice that received naïve T cells. Colon samples from host mice that received naïve T or naïve T + T_reg_ cells were collected at the endpoint. CW and BW were determined. Ratios of CW to BW (mg of colon weight per g of body weight) from both groups of host mice are shown. (**b**) Pathological examination by H&E staining. The inflammatory lesions were found in the colon and cecum, with a greatly thickened wall due to infiltration of inflammatory cells, and epithelial hyperplasia with crypt elongation. (**c**) The inflammatory status by tissue indicated are scored as described in Methods. Data from four repeated experiments are combined (naïve T, n = 14; naïve T + T_reg_, n = 14) and represented by Means ± SEM. (**p < 0.01, ****p < 0.0001).

We next conducted pathological examinations of both types of host mice to confirm whether the abdominal inflammation was correlated to the inflammation of the digestive tract. At the endpoint, the host mice were sacrificed. We found that the colons of mice that received naïve T cells became enlarged, thicker, and edematous, and they contained few intestinal components (Fig. 2a). The average colon weight (CW)/BW ratio of mice that received naïve T cells was 40.1 (mg of colon weight per g of body weight), which was twice that of mice that received naïve T + T_reg_ cells (21.0; *p* = 2.66 × 10^−5^). Increased spleen weight was also found in the hosts that received naïve T cells. As shown in Fig. S1a, spleen weight (SW)/BW ratio was significantly higher than that of the mice that received naïve T + T_reg_ cells (*p* = 0.018). Multifocal inflammatory lesions were found in the colon and cecum of mice that received naïve T cells, which had greatly thickened walls due to infiltration of inflammatory cells and epithelial hyperplasia with crypt elongation (Fig. 2b). Inflammatory lesions showed both chronic and chronic-active inflammation, as well as epithelial changes. The chronic inflammation consisted of infiltrates of lymphocytes and macrophages accompanied by small numbers of neutrophils and eosinophils. Occasional multinucleated giant cells were observed. Epithelial changes included hyperplasia, abnormal crypt morphology, and ulceration. Abnormal crypt morphology was characterized by marked crypt elongation, hypertrophy, and increased height of the epithelium lining with the glands. Ulceration was covered by an exudate of neutrophils and necrotic debris. Additional lesions consisted of goblet cell loss, erosion or ulceration, and crypt loss. Also observed was inflammation of the small intestine, consisting of mild to moderate infiltrate of lymphocytes and neutrophils in the lamina propria with apparent abnormal crypt morphology and goblet cell loss. The inflammatory statuses were scored as described in Methods and are shown in Fig. 2c. The pathological scores of the digestive tract were separated into several segments. Mice that received naïve T cells showed severe colitis (average score: 10–15), moderate to severe typhlitis (average score: 9.8), and mild to moderate enteritis (average score: 3.5). Taken together, the pathological scores of the mice that received naïve T cells were significantly higher than those of the mice that received naïve T + T_reg_ cells (proximal colon, *p* = 0.003; middle colon, *p* = 0.0001; distal colon, *p* = 0.0002; cecum, *p* = 0.0006)

### Luc-expressing T cell expanded in peripheral blood leukocytes (PBL) and induced cytokine production

**Figure 3:**
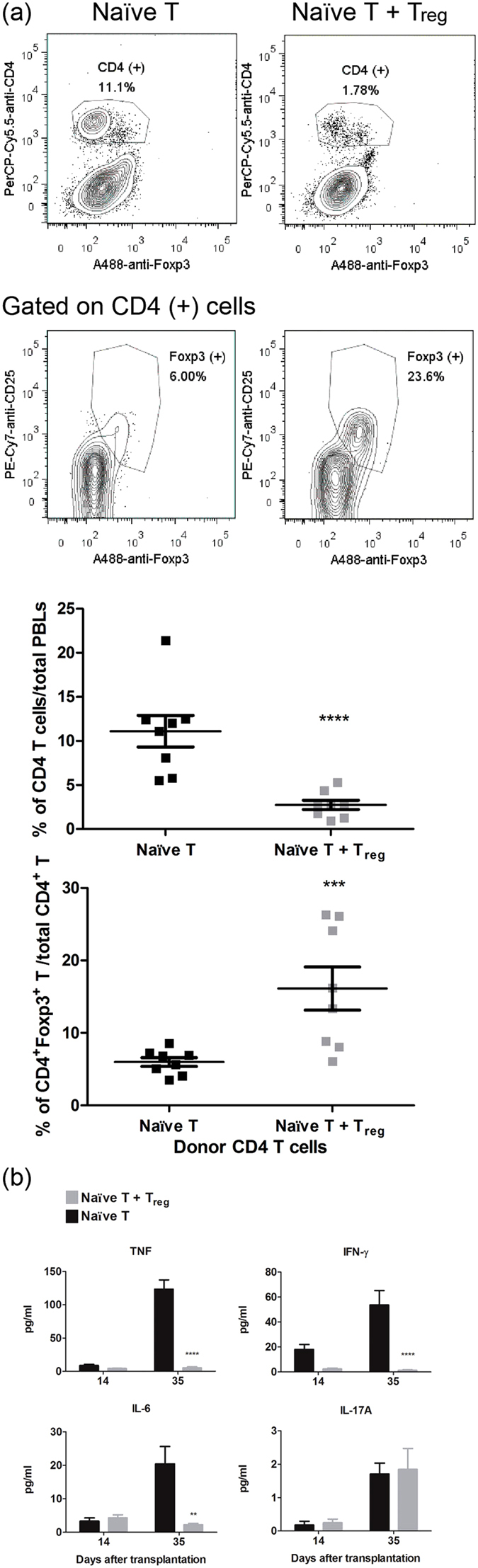
Luc-expressing T cell expanded in PBLs and cytokine production in sera. (**a**) Massive expansion of Luc-expressing T cell in PBLs. At D28 PAT, PBLs were collected and stained as described in Methods. Stained PBLs were then analyzed by flow cytometry. Percentages of CD4^+^ T cells to total PBLs and percentages of CD4^+^Foxp3^+^CD25^+^ T cells to total CD4^+^ T cells are shown. (**b**) Sera were collected at D14 and D35 PAT, and the serum cytokines as indicated were determined by CBA as described in Methods. Data from four repeated experiments are combined (naïve T, n = 14; naïve T + T_reg_, n = 14) and represented by Means ± SEM. **p < 0.01, ***p < 0.001, ****p < 0.0001).

We next analyzed the donor T cells in PBL and the serum cytokines to evaluate the inflammations of both host mice. PBLs from host mice at D28 PAT were analyzed by FACS (Fig. 3a). Massive expansion of donor CD4^+^ T cells (11.8% in total PBL on average) with few Foxp3^+^ T_reg_ phenotype donor T cells (5.98% in total CD4^+^ T cells on average) were found in mice that received naïve T cells. In contrast, fewer donor CD4^+^ T cells (2.7% in total PBL; *p* = 0.0005) with more T_reg_ phenotype donor T cells (16.1% in total CD4^+^ T cells; *p* = 0.005) were found in mice that received naïve T + T_reg_ cells. This phenomenon was observed not only in the PBLs but also in spleen cells and intraepithelial lymphocytes (IEL) of intestines at the endpoint (Figs S1b and S2).

To explore the cytokine production profiles of the Luc-expressing T cell-induced colitis model without animal sacrifice, we detected serum cytokines from host mice at D14 and D35 PAT (Fig. 3b). At D35 PAT, TNF, IFN-γ, and IL-6 were significantly higher in the serum of mice that received naïve T cells (123.4, 53.7, 20.4 pg/ml, respectively) than in that of mice that received naïve T + T_reg_ cells (5.3, 1.3, 2.2 pg/ml, respectively; TNF, *p* = 9.88 × 10^−7^; IFN-γ, *p* = 0.0003; IL-6, *p* = 0.005). Similar levels of serum IL-17A were detected in both groups at D35 PAT (~2 pg/ml). Very low serum IL-10 and IL-4 were detected in mice that received naïve T + T_reg_ cells (Fig. S3), and the levels were slightly, but not significantly, higher than in mice that received naïve T cells. All cytokines measured in sera maintained low concentrations, and there were no significant differences between the two groups at D14 PAT.

### Sensitive BLI analysis provided a better indicator than BW changes in the dexamethasone (DEXA) treatment trial

**Figure 4:**
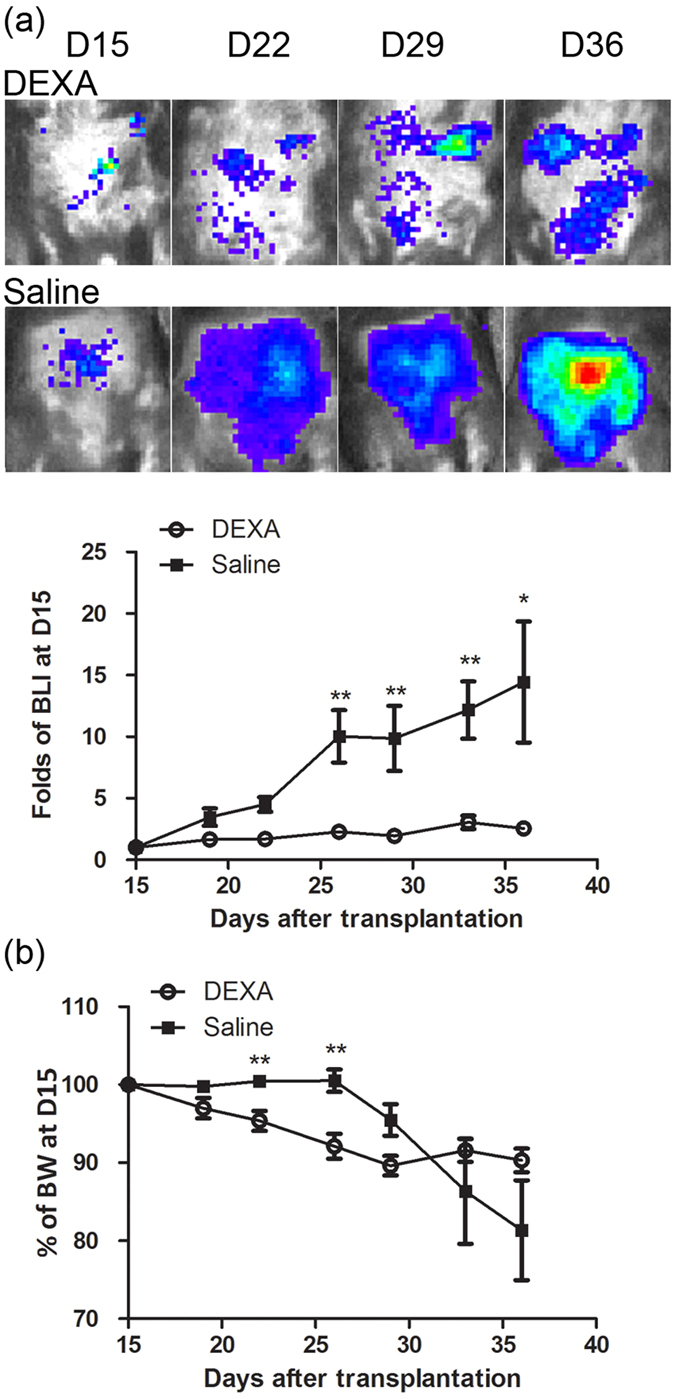
BLI analysis is more sensitive than BW measurement. (**a**) BLI analysis of Luc-expressing T cell-induced colitis model with or without DEXA treatment. Rag1-ko host mice received Luc-expressing naïve T cells and were analyzed with IVIS twice a week. Host mice were randomly separated into two groups at D15 PAT. One group was daily subcutaneously injected with 100 μg/mouse DEXA for three weeks, and the other, with normal saline. Folds of BLI at D15 PAT were calculated using total influx at each time point divided by the total influx at D15 PAT. (**b**) BW changes of host mice after DEXA-treatment were normalized by the BW at D15 PAT. Data from four repeated experiments are combined (DEXA, n = 16; saline, n = 12) and represented by Means ± SEM. (*p < 0.05, **p < 0.01).

One application of the naïve T cell-induced colitis model is for *in vivo* screening of immune-regulation drugs. We next applied DEXA treatment in the Luc-expressing naïve T cell-induced colitis model. Rag1-ko host mice that received naïve Luc-expressing T cells showed significant increases of BLI signals at D15 PAT and were randomly separated into two groups for DEXA treatment or saline control. BLI changes normalized by the BLI at D15 PAT are shown in Fig. 4a. While BLI signals continued to increase in the saline controls, the signals stopped increasing in the DEXA group for the duration of treatment. The BLI signals of the saline control group were significantly higher than those of the DEXA group at D26 PAT and thereafter (D26, *p* = 0.002; D29, *p* = 0.006; D33, *p* = 0.001; D36, *p* = 0.017). As for BW changes (Fig. 4b), the BW of DEXA-treated mice decreased from D15 to D29 PAT (89.6% of D15), and BW loss stopped until D36 PAT (90.3% of D15). The BW of saline control mice remained unchanged or slightly increased from D15 to D26 PAT and dramatically decreased to 81.3% at D36 PAT. This finding indicates that abdominal BLI determination is more sensitive and accurate than comparisons of BW measurements during therapeutic trials. To further explore whether the decrease in BW of DEXA-treated mice from D18 to D26 PAT was due to decreases in food or water intake, the average water intake and BW changes were concurrently recorded (Fig. S4). We indeed found that water intake was much lower in DEXA-treated mice than in the saline control mice (Fig. S4). This may be the reason why the DEXA-treated group exhibited BW loss despite not developing colitis. Again, BLI analysis is much more accurate than BW loss.

### DEXA treatment rescued the Luc-expressing T cell-induced colitis and enteritis

**Figure 5:**
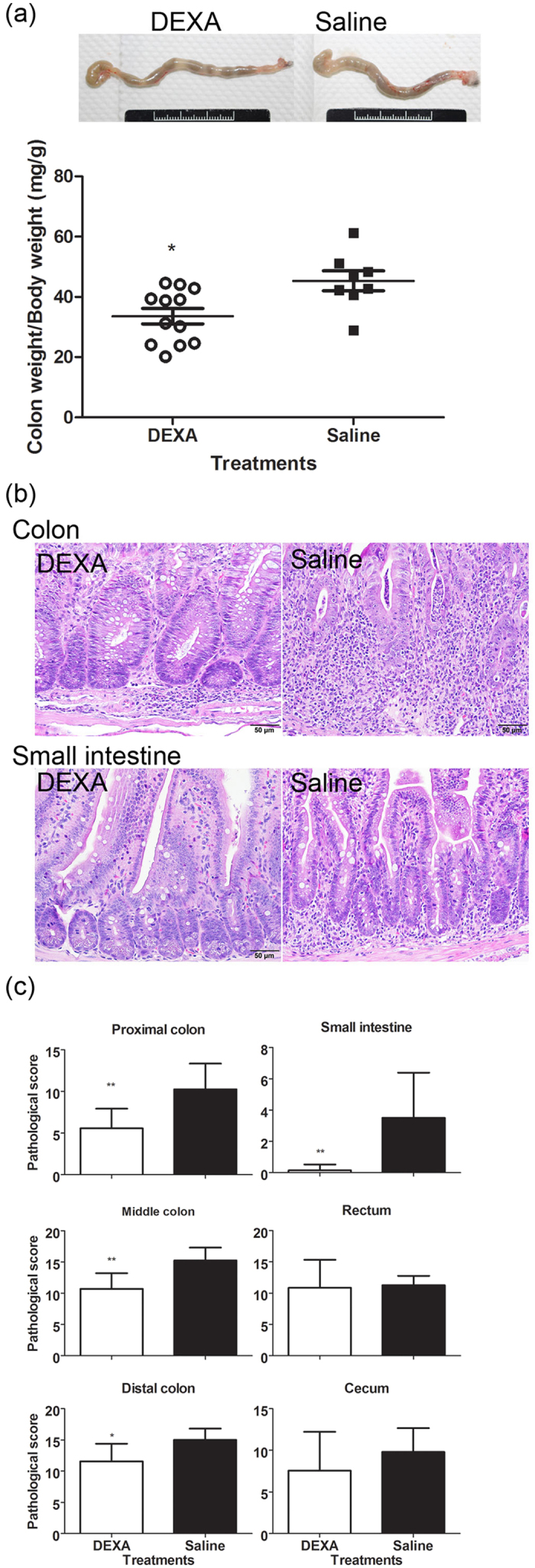
DEXA treatment rescued the Luc-expressing T cell-induced colitis and enteritis. (**a**) Reduction of swollen colons after DEXA treatment. Colon samples from mice that received naïve T cells at D0 and were treated with or without DEXA after D15 were collected at the endpoint. CW and BW were determined. Ratios of CW to BW from both group host mice were compared. (**b**,**c**) Pathological observations and scoring. Tissues from the mice indicated were prepared for pathological examination as described in Methods, and inflammatory status was scored. Data from four repeated experiments are combined (DEXA, n = 16; saline, n = 12) and represented by Means ± SEM. (*p < 0.05, **p < 0.01).

To verify the correlation between the abdominal BLI and actual status of colitis after DEXA-treatment, we analyzed the inflammation of the digestive tract by pathological observations. Although colons from DEXA-treated hosts were enlarged, they contained a few formed pellets inside. In contrast, colons from saline controls contained no or watery intestinal components (Fig. 5a). The average CW/BW ratio of DEXA-treated hosts was 33.6, which was significantly lower than that of saline control hosts (*p* = 0.0116). Similarly, the ratio of SW to BW of DEXA-treated hosts also significantly decreased (Fig. S5a, *p* = 0.035). Histopathological observations are shown in Fig. 5b. DEXA treatment reduced the wall thickness of the colons, infiltration of inflammatory cells, and regenerated crypts. In addition, DEXA treatment fully rescued the inflammation of the small intestine. The pathological scores of DEXA-treated host mice in Fig. 5c indicated that inflammation was mild to moderate in the cecum and proximal colon (average scores: 7.6 and 5.6), and moderate to severe in the middle colon to rectum (average score: 10.7–11.5). Taken together, the pathological scores of DEXA-treated host mice were significantly lower than saline control hosts (proximal colon, *p* = 0.02; middle colon, *p* = 0.006; distal colon, *p* = 0.02).

### DEXA treatment reduced donor T cells survival and induced suppressive cytokine production in sera

**Figure 6:**
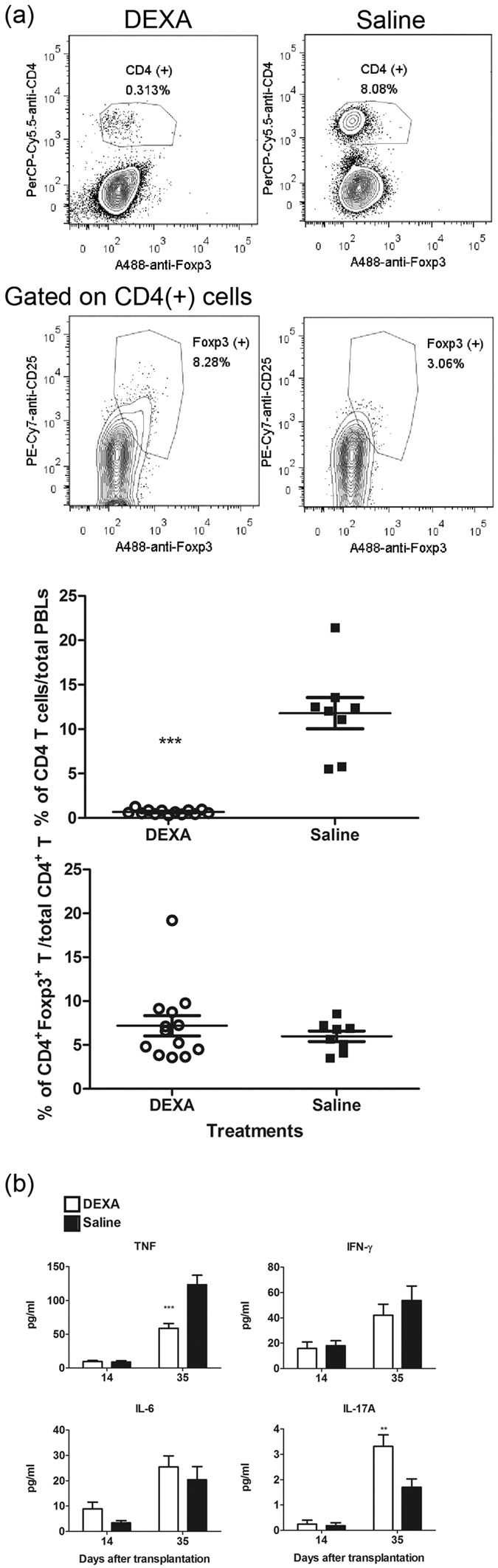
DEXA treatment reduced donor T cells survival and induced suppressive cytokine production in sera. (**a**) Reduction of Luc-expressing T cell expansion in PBLs from Rag1-ko mice that received naïve T cells and were treated with DEXA for three weeks. At D28 PAT, PBLs were s stained as described in Methods, and then analyzed by flow cytometry. Percentages of CD4^+^ T cells in total PBL and percentages of CD4^+^Foxp3^+^CD25^+^ T cells in CD4^+^ T cells are shown. (**b**) Sera were collected at D14 and D35 PAT and analyzed for serum TNF, IFN-γ, IL-6, and IL-17A production by CBA kits as described in Methods. Data from four repeated experiments are combined (DEXA, n = 16; saline, n = 12) and represented by Means ± SEM. (**p < 0.01, ***p < 0.001).

We further analyzed the donor T cells in PBLs and the serum cytokines after DEXA-treatment. PBLs from host mice at D28 PAT (14 days after DEXA treatment) were analyzed by FACS (Fig. 6a). Percentages of donor CD4^+^ T cells to total PBLs from DEXA-treated host mice significantly fell to 0.7%, which was 6.0% of that from saline control mice (*p* = 0.0001). Similar results were also found in spleen cells and IEL of the intestines at the endpoint (Figs S5b and S6). In addition, the percentages of Foxp3^+^ T_reg_ phenotype CD4^+^ T cells were similar in PBLs and spleen, but they were higher in the IEL in DEXA-treated than in control saline hosts. As a result, DEXA inhibited colitis induction may have been due to reduced donor CD4^+^ T cell survival in the Rag1-ko host.

To explore the cytokine production profiles of Luc-expressing T cell-induced colitis after DEXA treatment, we detected serum cytokines from host mice at D14 (before therapy) and D35 (after therapy) PAT (Fig. 6b). Only TNF significantly decreased, by two folds, in the serum from DEXA-treated host mice as compared with the saline controls (*p* = 0.0003). Moreover, DEXA treatment did not reduce serum IFN-γ or IL-6 levels. Induced serum IL-17A levels were low in DEXA-treated mice, but two folds higher than in saline control mice (*p* = 0.004). Induced serum IL-10 levels were also low (Fig. S7) but significantly higher than in saline control host mice (*p* = 0.039). Similarly, very low serum IL-4 concentrations (Fig. S7) were detected in DEXA-treated host mice, though they were a slightly higher than those of saline control host mice, with no statistically significant differences.

## Discussion

In this study, we established a modified autoimmune colitis model using Luc-expressing naïve T cells and BLI analysis. BLI analysis appeared to be more sensitive and accurate for detecting the onset of autoimmune colitis (Fig. 1) and had very good correlation with pathological observations (Fig. 2). FACS analysis results showed massive expansion of donor T cells with a low frequency of T_reg_ phenotype cells and high TNF, IL-6, and IFN-γ production in mice that received naïve T cells (Fig. 3). Furthermore, we applied this model in a pre-clinical trial with DEXA therapy. This modified model was more sensitive and accurate for judging the drug efficacy of the CD model by BLI analysis than by BW loss (Fig. 4). Pathological observations (Fig. 5) showed that the recovery of the upper intestine (small intestine, cecum and proximal colon) was faster than that of the lower intestine (middle colon, distal colon and rectum) after DEXA treatment. However, DEXA treatment significantly reduced only TNF, and it increased IL-17A levels in sera (Fig. 6). Taken together, these results show that our modified model can be used to study the efficacy of immune-regulation drugs and immune gene-regulation in the autoimmune colitis model.

The BLI intensities showed a large difference of at least one order of magnitude, not only between mice that received naïve T and those that received naïve T + T_reg_ cells but also between DEXA-treated and saline control (untreated) mice (Figs 1 and 4). BLI is a more sensitive indicator than BW loss. This high sensitivity of an *in vivo* luciferase reporting assay provides the benefit of detection of onset of autoimmune colitis at least 7 days earlier (Figs 1 and 4). This early detection should be very useful for pre-clinical trials of this CD model and may allow the application of therapeutic strategies as early as possible in animals exhibiting severe diarrhea. Application of this Luc-expressing T cell-induced colitis model using the standard anti-inflammatory drug DEXA revealed this advantage clearly. BLI intensity did not increase in host mice immediately after DEXA administration (Fig. 4) and was significantly lower than in saline control host mice at D26 PAT and thereafter.

One of the successful factors of this model is the stable luciferase expression in Luc-expressing T cells. The human ubiquitin promoter provides a stable expression of luciferase in our Luc Tg. T cells. Therefore, the increase in BLI intensity indicates the expansion of donor Luc-expressing T cells *in vivo*. This expansion suggests that the Luc-expressing T cells may be used in other experiments for quantification of T cell numbers *in vivo*.

In both mice that received naïve T and those that received naïve T + T_reg_ cells, BLI intensity increased over time by one order of magnitude before D14 PAT (Fig. 1b). Two possibilities can explain this increase. First, homeostatic expansion in Rag1-ko mice may have occurred during the first 14 days after transfer. Second, the inflammation responses specific to self-antigens may have started immediately after transfer, and the immune regulatory functions of T_reg_ cells did not suppress the autoimmune inflammation as quickly as the response happened. However, serum cytokine levels were very low at D14 PAT (Fig. 3b). In addition, the frequency of donor T cells in PBL was very low in both groups of mice at D14 PAT as compared with the frequency at D28 (Fig. 3a). These differences indicate that homeostatic expansion of donor T cells occurred during the first 14 days PAT. Our finding also agrees with the homeostatic proliferation of transferred T cells in syngeneic lymphopenia host mice^10^,^11^,^12^. Homeostatic expansion of T cells in lymphopenia hosts in the early stage is very important in the development of systemic autoimmune disease^12^. Polyclonal expansion of donor T cells in lymphopenia host mice contains self-reactive T cells. These self-reactive T cells are then activated and induced autoimmune colitis in host mice. This suggests that the high sensitivity of BLI analysis also clearly provides evidence of the homeostatic proliferation of donor T cells *in vivo*, although very few donor T cells in PBL were detected by FACS.

Based on the pro-inflammatory cytokine profile in IBD patients, autoimmune colitis is a Th1 (IL-12/IFN-γ) and Th17 (IL-17 and IL-23)-mediated response^13^,^14^. However, IL-17A has been reported to protect T cell-mediated intestinal inflammation by inducing atypical M2-like macrophages^15^,^16^. IL-6 has also been detected in IBD patients and colitis mice^17^,^18^. IL-6 is majorly produced by macrophages and is known as a key factor in cooperation with TGF-β to induce Th17 cells. In addition, TNFs is also elevated in the serum, stool, and mucosa of IBD patients and mouse models^19^,^20^,^21^. Although TNF can be produced by both lymphocytes and non-lymphocytes, TNF derived from non-lymphoid cells in host mice is required for induction of colitis^22^. Our cytokine profile data indicated that Luc-expressing naïve T cells induced IFN-γ, Th17, TNF, and IL-6 elevation at the endpoint, which is consistent with previous findings. Interestingly, IL-17A was also detected in mice that received naïve T + T_reg_ cells (Fig. 3b). There are two possible reasons for this. One is that naïve T + T_reg_ donor cells might have undergone a balanced immune response, so high pre-inflammatory IL-17A was detected without elevation of other causative cytokines. The other reason could be that the source of this IL-17A was a subset of IL-17-producing CD4^+^ Foxp3^+^ CD25^+^ regulatory T cells, which has been addressed in the human periphery^23^. Moreover, IL-17A has also been reported to play a protective role in T cell-mediated intestinal inflammation^15^.

Immune depression by DEXA treatment is extensive. DEXA induces apoptosis of T cells in the spleen and thymus^24^,^25^. DEXA promotes IL-4, IL-10, and IL-13 Th2 cytokines and reduces synthesis of IFN-γ and TNF-α during *in vitro* activation of CD4^+^ T cells^26^. DEXA also induces apoptosis of macrophages and regulates the functions of macrophages by depression of TNF-α releasability and phagocytosis^27^. DEXA treatment significantly decreased TNF (known to be mainly produced by monocytes) and slightly reduced IFN-γ (known to be mainly produced by T cells) in this trial (Fig. 6). These effects indicate that DEXA treatment inhibited both innate and adoptive immune responses in this trial.

DEXA treatment reduced BLI (Fig. 4) and pro-inflammatory TNF (Fig. 6), and induced anti-inflammatory IL-17A (Fig. 6) and IL-10 (Fig. S7). However, serum IL-6 and IFN-γ levels remained similar to those in saline control mice (Fig. 6). Collectively, 3-wk-DEXA treatment thus generated a transition status from the inflammatory stage to the quiescent stage. IL-17 has been reported to stimulate the differentiation of human anti-inflammatory M2 macrophages in response to IL-10 and DEXA^28^. Our finding is that increased IL-6 in DEXA-treated mice may therefore be produced by M2 macrophages, which were previously reported to be the source of IL-6^29^,^30^. In addition, we noted that neutrophils increased in PBLs after DEXA treatment (Fig. S8), which is consistent with a previous report^31^. As a result, increased IL-6 and IFN-γ may also be secreted by the neutrophils, which were found to be capable of producing IL-6 and IFN-γ^32^.

One of the best-known side effects of long-term glucocorticoid treatment is increased BW. However, the BW of host mice decreased after DEXA treatment (Fig. 4b, D19 to D29). This decrease occurred soon after starting DEXA treatment, before the onset period of glucocorticoid-induced BW increases. Thus, the BW increase was not the glucocorticoid-induced side effect. In rats, glucocorticoid treatments decreased BW and food intake^33^. Thus, it indicated that DEXA might also decrease appetite and BW in autoimmune colitis mice. According to animal welfare practices, mice cannot be single-housed mice without good reason, so it is hard to measure the food and water intake of single-housed mice. In addition, it is known that adequate intake significantly influences the BW of laboratory mice^34^. Therefore, we recorded the BW changes and evaluated the average water intake of the group-housed mice. The results indicated that the DEXA-treated mice reduced their water intake greatly as compared to the saline control mice (Fig. S4), and their BW concurrently decreased during the same period. Although the saline control mice also decreased their water intake from D18 to D26 PAT, their BW did not decrease. Based on the increased BLI in saline control mice, one possible reason could be that the inflammation of the gastrointestinal tract increased the weight of the saline control mice. Therefore, the BW loss related to low water intake was offset by the increase in weight caused by inflammation of the gastrointestinal tract.

In recent years, animal welfare has become a very important issue in the animal experiments. While the experimental animals lost more than 20% BW, the humane endpoints should be considered. Thus, our model can provide a larger window for observation of the progression of this autoimmune colitis model. In accordance with the 3Rs principle, we strive to reduce animal sacrifices. With the modified model and method, the disease progression can be known well, with no need to sacrifice any mice in the early stage.

In summary, we established a modified transferred T cell-induced colitis mouse model by transferring Luc-expressing naive T cells and measuring the BLI by IVIS. This modified model can detect the onset of abdominal inflammation earlier than, and has very good correlation with the pathological and immunological disease progressions of, the traditional transferred T cell-induced colitis mouse model. It will be a useful tool for researchers of autoimmune colitis.

## Methods

### Mice

C57BL/6-Tg(UBC-mKate-Luc) mice (Luc Tg. mice) were generated by Gene Engineering Murine Model Service (GEMMS) and deposited in the Rodent Model Resource Center (RMRC). B6.129S7-Rag1^tm1Mom^/J mice (Rag1-ko) were obtained from The Jackson Laboratory. Both Rag1-ko and Luc Tg. mice were bred and held in the animal rooms of National Laboratory Animal Center (NLAC). C57BL/6JNarl mice were purchased from NLAC. Hemizygous Luc Tg. mice were bred with wildtype B6 mice. The genomic DNA from Luc Tg. offspring were collected and screened by PCR (Fig. S9). Luc4637F (Sense, TCACGCAGGCAGTTCTATGAGG) and Luc5147R (Anti-sense, GAGGTGGACATCACTTACGCTGAG) primers were applied to amplify a 510-bp fragment of the luciferase transgene. Mice were used at 8–16 weeks of age for the experiments. The animal protocol was approved by the Institutional Animal Care and Use Committee (IACUC) of NLAC (IACUC2012M12). The animal protocol was carried out in “accordance” with the approved guidelines. Post approval monitoring (PAM) programs were implemented during the research.

### Establishment of human IBD animal model by Luc-expressing T cells

Luc Tg. or B6 mice were sacrificed and spleens were collected. The spleens were gently homogenized by forceps to collect single cell suspensions, treated with ACK buffer (0.83% NH_4_CL, 0.1% KHCO_3_) to lyse RBC, and stained with FITC-anti-CD4 (Clone: GK1.5), PE-anti-CD45RB (Clone: 16A), and APC-anti-CD25 (Clone: 7D4). Luc-expressing CD4^+^ CD45RB^hi^CD25^−^ naïve T cells and B6 CD4^+^ CD45RB^lo^CD25^+^ T cells (T_reg_) were purified by FACSAria (BD Bioscience). The purity of naïve T cells was >95%. Luc-expressing naïve CD4^+^ T cells were then transferred into Rag1-ko mice (6 × 10^5^ cells/mouse). The control Rag1-ko host mice received Luc-expressing naïve T (6 × 10^5^ cells/mouse) and B6 T_reg_ cells (3–4 × 10^5^ cells/mouse). Body weights were recorded twice a week. DEXA treatment trial was performed using subcutaneous injection of DEXA (100 μg/100 μl/mouse) or 100 μl normal saline (Saline control) daily for 3 weeks. When loss of body weight exceeded 20% after transfer, the host mice were sacrificed. All tissues were subjected to pathological observations and sera were collected for cytokine profile analysis at endpoint (D36 PAT).

### BLI analysis

The BLI analyses of each host mouse were conducted twice per week. The host mouse was injected intraperitoneally with 200 μl D-luciferin (15 mg/ml, Cat. L-123–250, GoldBio). After 10 minutes, the mouse was then anesthetized with isoflurane (3% vaporized in O_2_) and BLI analyzed by IVIS (IVIS 100, Caliper). The collected images were measured (Total photon flux, photons/sec) from a fixed region-of-interest (ROI) over the abdomen using Living Image software (Caliper).

### Immunological analysis

Peripheral blood from host mice 4 wk PAT was collected from facial veins (Submandibular) using Golden Lancets (4 mm, Medipoint Inc.), and spleens from host mice at endpoint were collected. Sample RBC was lysed by ACK buffer. Peripheral blood leukocytes (PBLs) and spleen cells were stained with surface markers PerCP-Cy5.5-anti-CD4 (Clone: RM4-5, Cat. 550954, BD Pharmingen) and PE-Cy7-anti-CD25 (Clone: PC61, Cat. 561780, BD Pharmingen). The stained cells were fixed with the Intracellular Fixation & Permeabilization Buffer Set (Cat. 88-8824-00, eBioscence), stained with Alexa Fluor^®^ 488 (A488) anti-Foxp3 antibody (Clone: MF23, Cat. 560403, BD Pharmingen), and then analyzed by FACS LSRFortessa (BD). Collected data were analyzed by FlowJo software (StarTree). Sera from host mice at D14 (the start point to detect BLI differences between naïve T and naïve T + T_reg_ groups) and D35 (the day before endpoint) were collected. The serum IL-2, IL-4, IL-6, IL-10, IL-17A, TNF, and IFN-γ analyses were measured by Th1/Th2/Th17 Cytokine Kit (Cat. 560485, BD Cytometric Bead Array). All antibodies and cytokine bead array kits were purchased from BD Bioscience.

### Pathological examination

Tissues from host mice at endpoint were collected for pathological observations. Colon weight (CW), spleen weight (SW), and body weight (BW) were determined. The ratios of CW (mg) to BW (g) or SW (mg) to BW (g) were calculated and used as an indicator of disease progression. Histological examination for H&E staining was performed according to standard procedures. Three sections of each tissue sample were made from each host mouse. One to two samples were examined from each of the following digestive tract tissues: duodenum, cecum, proximal colon, middle colon, distal colon, and rectum. Intestinal samples were sectioned transversely. Other tissues examined with gross lesions included spleen, liver, and/or lungs. Tissues were embedded in paraffin, sectioned to 3 μm thickness, and stained with hematoxylin and eosin (H&E). The severity of inflammation was scored as follows: X (no significant lesions); extent of inflammation (0, none; 1, lamina propria; 2, submucosal; 3, transmural); degree of inflammation (0, none; 1, mild; 2, moderate; 3, severe); abnormal crypt morphology (0, none; 1, mild; 2, moderate; 3, severe); neutrophil infiltration (0, none; 1, mild; 2, moderate; 3, moderate-severe; 4, severe); goblet cell loss (0, none; 1, moderate; 2, severe); mucosal erosions or ulceration (0, none; 1, lesion present); and crypt abscesses (0, none; 1, lesion present). The maximum score was 17.

### Statistics

Analyses of BLI, BW changes, cytokine production and pathological scores of inflammatory digestive tract were performed using one-tailed Student’s *t* test. All statistics were calculated using GraphPad Prism version 5 for Windows. The results were statistically significant when *p* < 0.05.

## Additional Information

**How to cite this article**: Chen, Y.-L. *et al.* Early Detection of T cell Transfer-induced Autoimmune Colitis by In Vivo Imaging System. *Sci. Rep.* **6**, 35635; doi: 10.1038/srep35635 (2016).

## Supplementary Material

Supplementary Information
