# Supplementary material for: Early Detection of T cell Transfer-induced Autoimmune Colitis by In Vivo Imaging System
Source: Sci Rep. 2016 Oct 20;6:35635. doi: 10.1038/srep35635 (PMC5071899; doi:10.1038/srep35635)
Supplement: Supplementary Information [file srep35635-s1.doc]

Title: Early Detection of T cell Transfer-induced Autoimmune Colitis by In Vivo Imaging System

Authors: Yu-Ling Chen†^1^; Yi-Ting Chen^†2^; Cheng-Feng Lo^1^; Ching-I Hsieh^1^; Shang-Yi Chiu^1^; Chang-Yen Wu^1^; Yu-Shan Yeh^1^; Shu-Hsuan Hung^1^; Po-Hao Cheng^1^; Yu-Hsuan Su^1^; Si-Tse Jiang^1^, Hsian-Jean Chin^1^; Yu-Chia Su*^1^

Affiliations: ^1^National Laboratory Animal Center, National Applied Research Laboratories, Taipei, Taiwan; ^2^Institute of Molecular Biology, Academia Sinica, Taipei, Taiwan;

Phone number: 886-2-27895861

Fax number: 886-2-27895577

† Yu-Ling Chen and Yi-Ting Chen contributed equally to this study.

* Address correspondence and reprint requests to Dr. Yu-Chia Su, National Laboratory Animal Center, National Applied Research Laboratories, No.128, Sec. 2, Academia Rd., Nangang Dist., Taipei City 11529, Taiwan (R.O.C.); E-mail addresses: ycsu@nlac.narl.org.tw


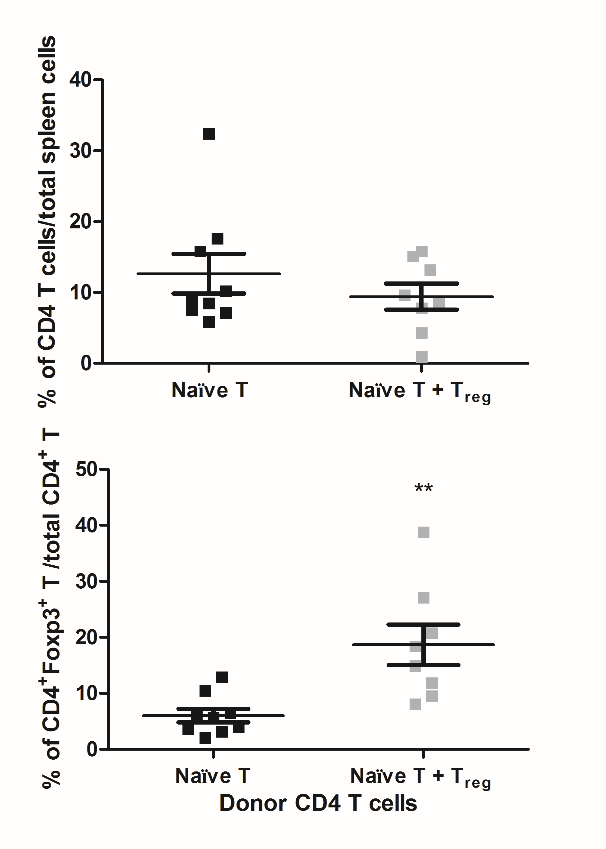

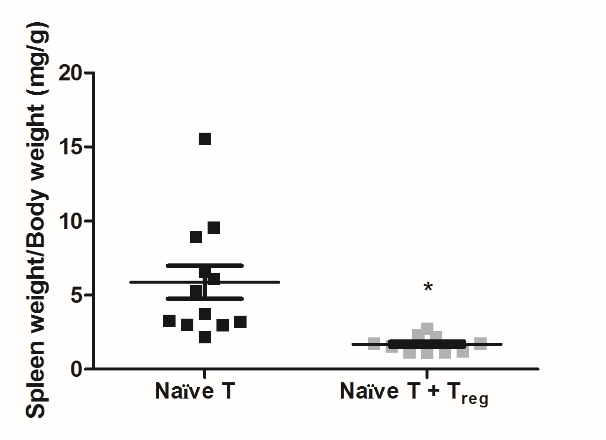


**Figure S1.  Adoptive transfer of Luc-expressing T cells expanded in spleen.** (a) Enlargement of spleens from host mice received naïve T cells. Spleen samples from host mice received naïve T or naïve T + T_reg_ cells were collected at the endpoint. Spleen weight (SW) and BW were determined. Ratios of SW to BW from both group hosts are compared. (naïve T, n = 12; naïve T + T_reg_, n =14). (b) FACS analysis of donor T cells in the spleen. The spleen cells were stained with PE-Cy7-anti-CD25, PerCP-Cy5.5-anti-CD4, and A488-anti-Foxp3, and then analyzed by flow cytometry. Percentages of CD4^+^ T cells to total spleen cells and percentages of CD4^+^Foxp3^+^ T cells to total CD4^+^ T cells are shown. Data from two repeated experiments are combined (naïve T, n = 9; naïve T + T_reg_, n =8) and represented by Means ± SEM. (**p* < 0.05,***p* < 0.01).

**Naïve T + T_reg_**

**Naïve T**

Supplemental Figure 1

**(a)**

**(b)**


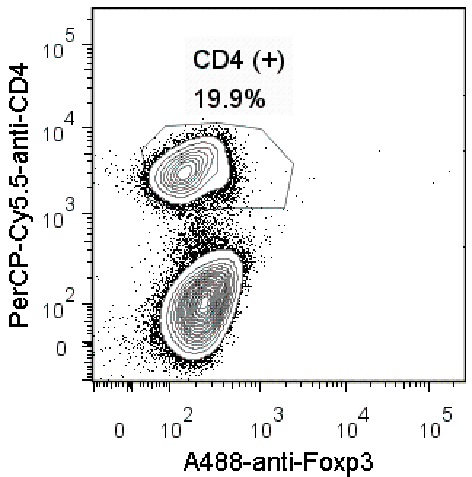

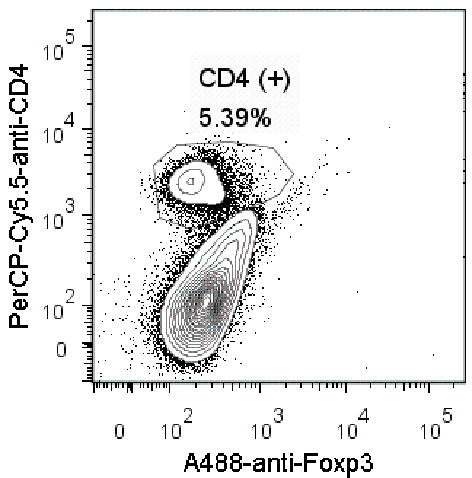

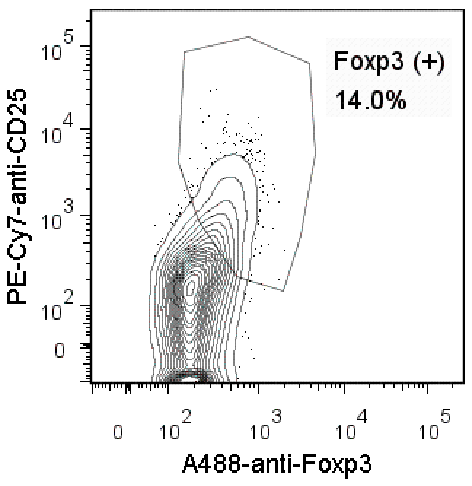

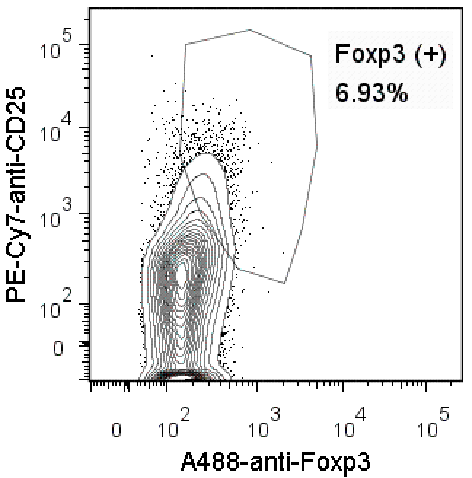


**Figure S2.  Co-transfer of naïve and T_reg_ T cells did not result in accumulation of donor cells in the intraepithelial lymphocytes (IEL).** Intestinal samples from host mice received naïve T or naïve T + T_reg_ cells were collected at the endpoint as shown in figure 2. The single cells were harvested by GentlMACS without any digestive enzymes. IELs were enriched with 40/70 percoll gradient purification and stained with PE-Cy7-anti-CD25, PerCP-Cy5.5-anti-CD4, and A488-anti-Foxp3. Stained IELs were then analyzed by flow cytometry. Percentages of CD4^+^ T cells to total IELs and percentages of CD4^+^Foxp3^+^ T cells to total CD4^+^ T cells are shown. Data from two repeated experiments are combined (naïve T, n = 10; naïve T + T_reg_, n =8) and represented by Means ± SEM. (****p* < 0.001, *****p* < 0.0001).

**Naïve T + T_reg_**

**Naïve T**

Supplemental Figure 2


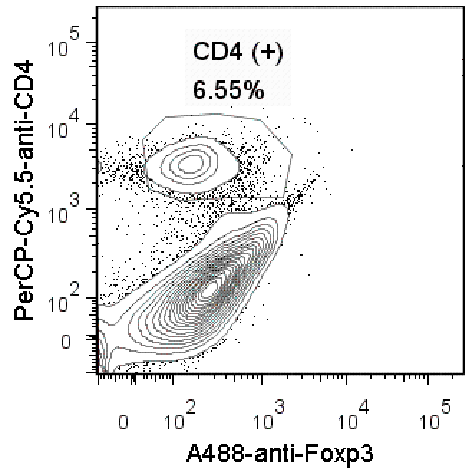

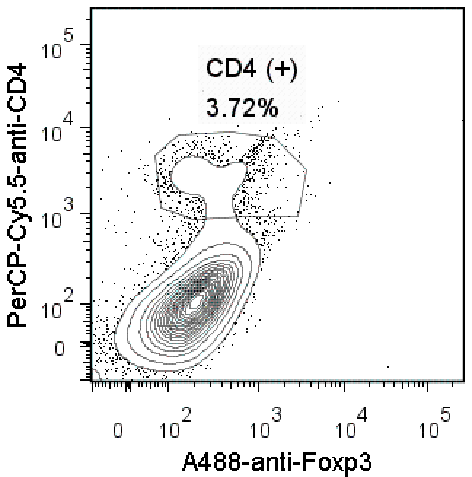

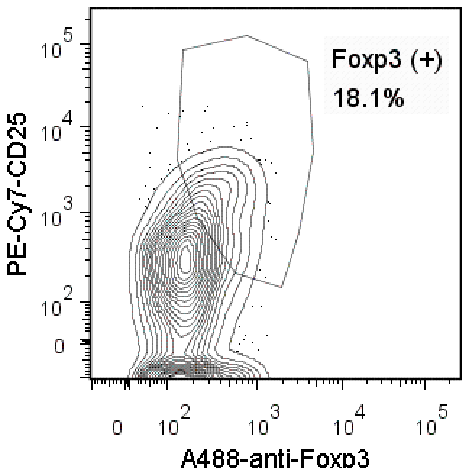

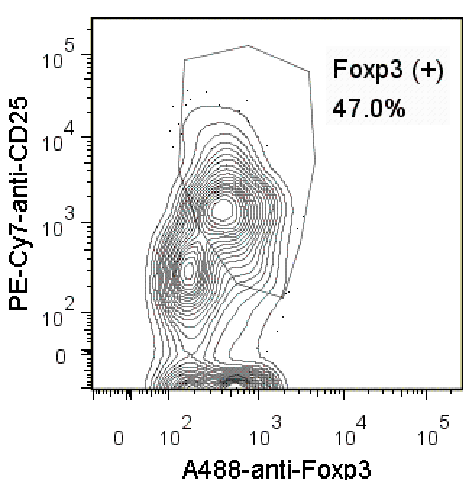

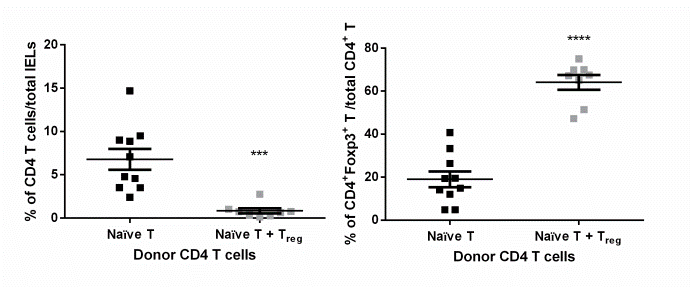

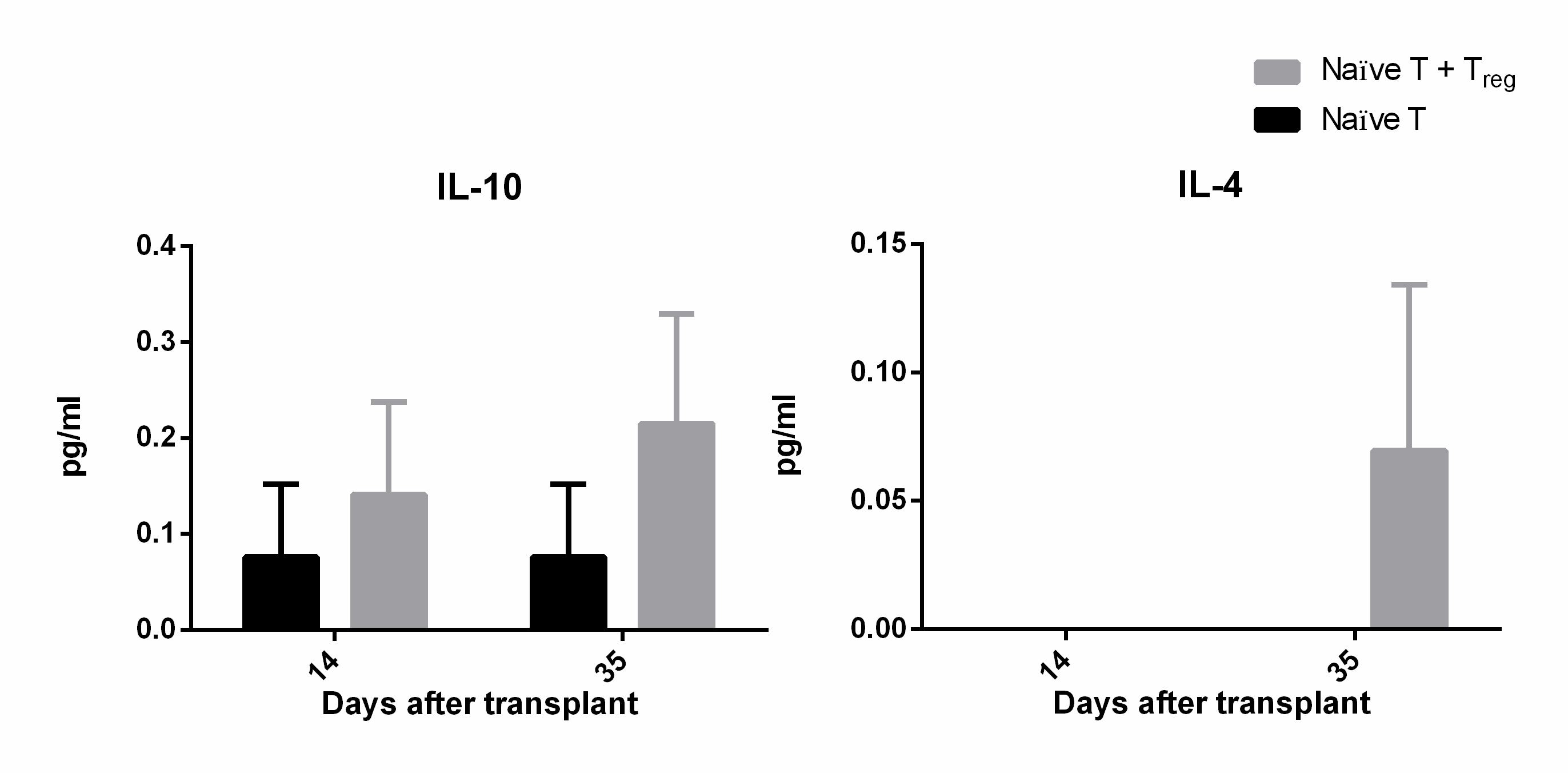


Supplemental Figure 3

**Figure S3. Induced low level IL-10 and IL-4 were detected in sera from host mice that received naïve T + T_reg_ cells.** Sera were collected at D14 and D35 PAT as described in Figure 3, and the IL-10 and IL-4 as indicated were determined by CBA as described in Methods. Data from four repeated experiments are combined (naïve T, n = 14; naïve T + T_reg_, n =14) and represented by Means ± SEM).


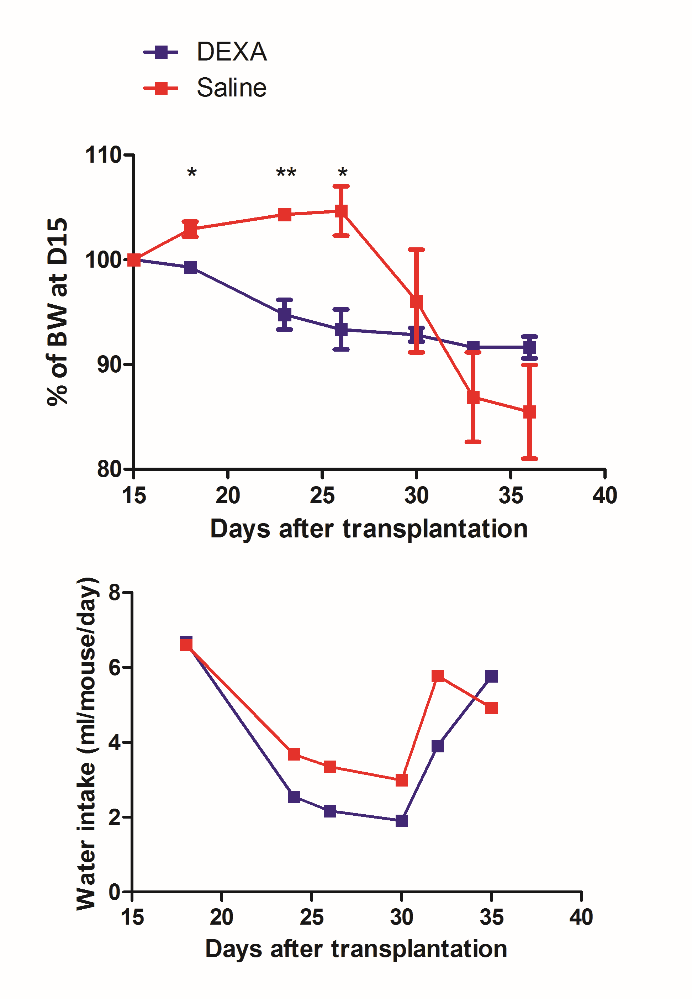


**Figure S4. Body weight (BW) changes of DEXA-treated mice are correlated with reduction of daily water intake.** (a) BW changes of host mice after DEXA-treatment were normalized by the BW at D15 PAT. Data are shown and are presented by Means ± SD. (DEXA, n = 5; saline, n = 5; **p*< 0.05, ***p*< 0.01). The BW of DEXA-treated mice decreased from D18 to D26 and then remained stable from D26 to D36 PAT. In contrast, the BW of saline control mice slightly increased from D15 to D26 PAT (4.5% increase) before dramatically decreasing to 88.9% at D36 PAT. (b) The mice shown in panel (a) were group-housed. Water bottles were weighed daily after D15 PAT. Daily differences in water bottle weights were calculated and then normalized by the number of mice in the cage (water intake: ml/mouse/day). Only data at indicated time points compared to panel (a) are shown. The daily water intake of both DEXA-treated and saline control mice decreased after treatment *via* intraperitoneal injection began. That of saline control mice decreased from D17 (6.6 ml/mouse/day) to D24 (3.7 ml/mouse/day) before recovering at D32 (5.8 ml/mouse/day). That of DEXA-treated mice decreased more significantly from D17 (6.7 ml/mouse/day) to D24 (2.3 ml/mouse/day) before partially recovering at D32 (3.9 ml/mouse/day).

**(a)**

**(b)**

Supplemental Figure 4


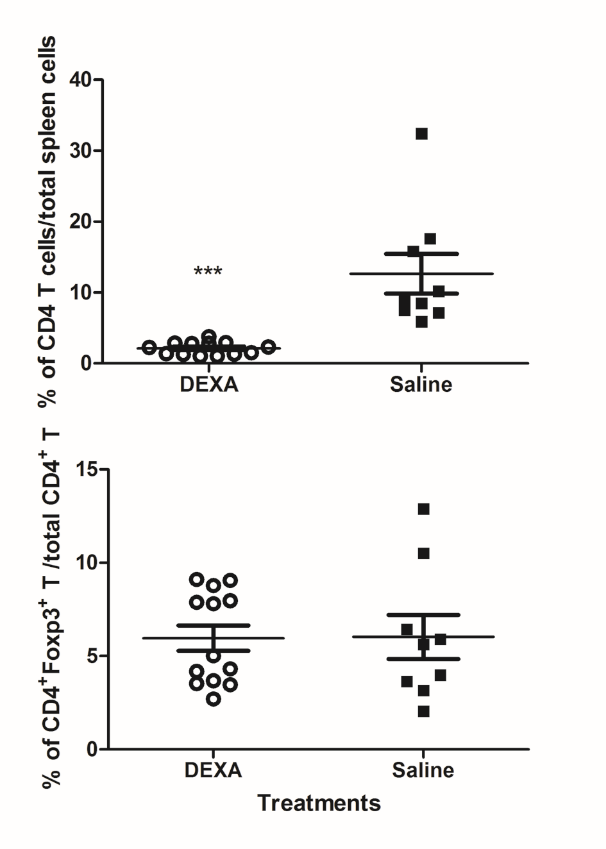


**Figure S5.   DEXA treatment decreased percentages of donor T cells in total spleen cells.** (a) DEXA-treatment reduced SW of host mice received Luc-expressing naïve T cells. The host mice were randomly separated into two groups by BLI analysis and treated with DEXA daily described as in Figure 4. At the endpoint, spleen samples from host mice were collected. SW and BW were determined. Ratios of SW to BW from both group hosts are compared. (DEXA, n = 13; saline, n = 8). (b) Decrease of Luc-expressing T cell in the spleen after DEXA treatment. Spleen cells were harvested as described previously. The spleen cells were stained with PE-Cy7-anti-CD25, PerCP-Cy5.5-anti-CD4, and A488-anti-Foxp3, and then subjected to FACS analysis. Percentages of CD4^+^ T cells to total spleen cells and percentages of CD4^+^Foxp3^+^ T cells to total CD4^+^ T cells are shown. Data from two repeated experiments are combined (DEXA, n = 13; saline, n = 10) and represented by Means ± SEM. (**p* < 0.05, ****p* < 0.001).

**DEXA**

**Saline**

Supplemental Figure 5

**(b)**


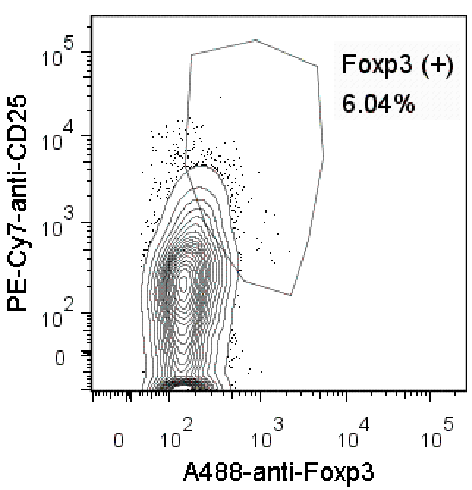

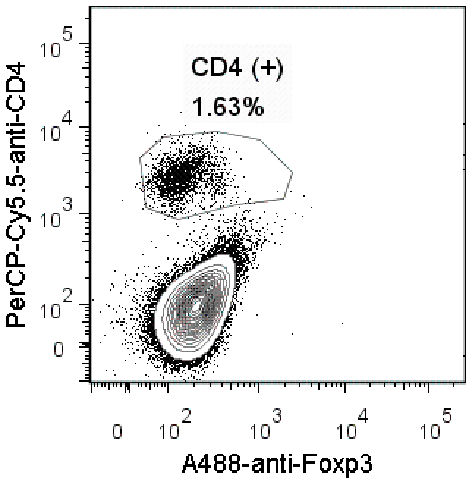

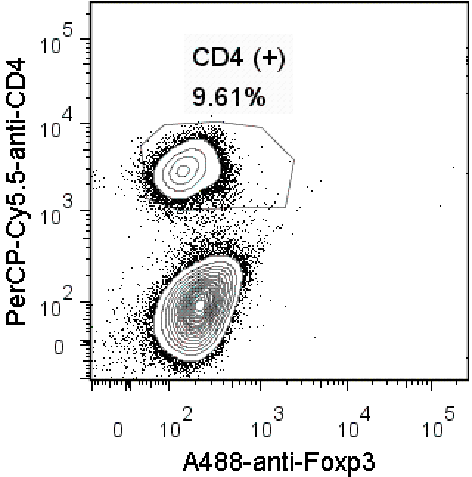

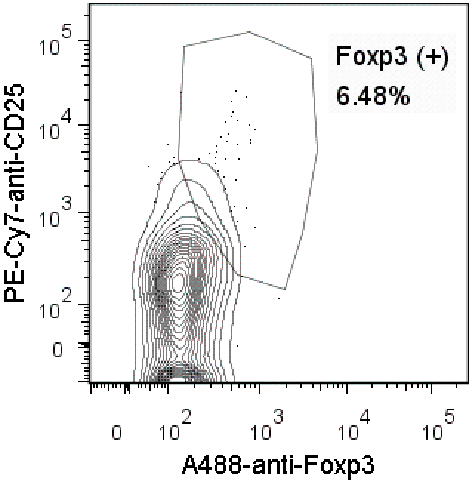


**(a)**

**DEXA**

**Saline**

Supplemental Figure 6

**Figure S6. DEXA-treatment decreased percentages of donor T cells in IELs.** Intestinal samples from host mice were collected at the endpoint as shown in figure 5. IELs were enriched and stained as described in figure S3. Stained IELs were then analyzed by flow cytometry. Percentages of CD4^+^ T cells to total IELs and percentages of CD4^+^Foxp3^+^ T cells to total CD4^+^ T cells are shown. Data from two repeated experiments are combined (DEXA, n = 13; saline, n = 10) and represented by Means ± SEM. (***p* < 0.01).


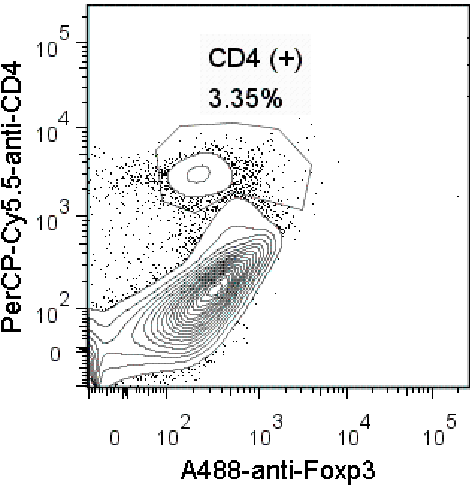

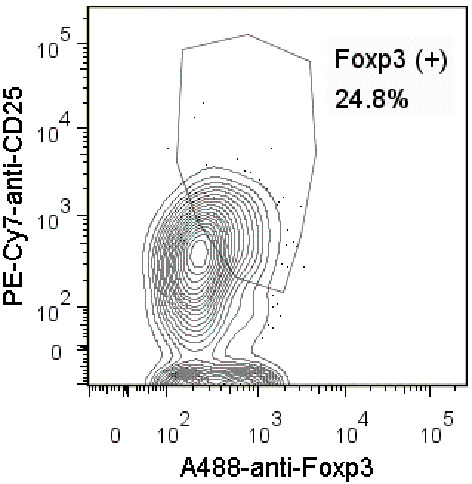

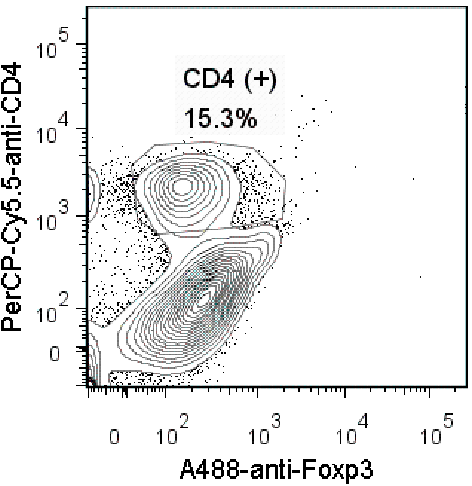

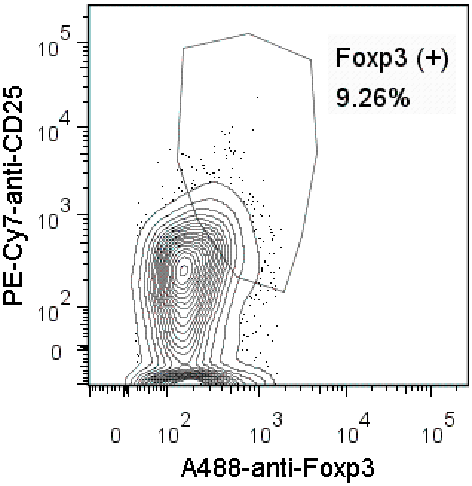

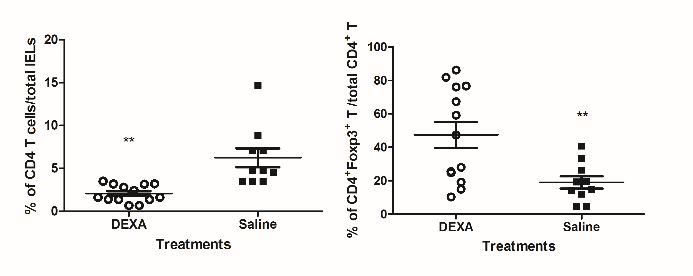


Supplemental Figure 7

**Figure S7. Higher level IL-10 and IL-4 were measured in sera from DEXA-treated mice.** Sera were collected at D14 and D35 PAT as described in Figure 6, and the IL-10 and IL-4 as indicated were determined by CBA as described in Methods. Data from four repeated experiments are combined (DEXA, n = 16; saline, n =12) and represented by Means ± SEM. *p < 0.05).


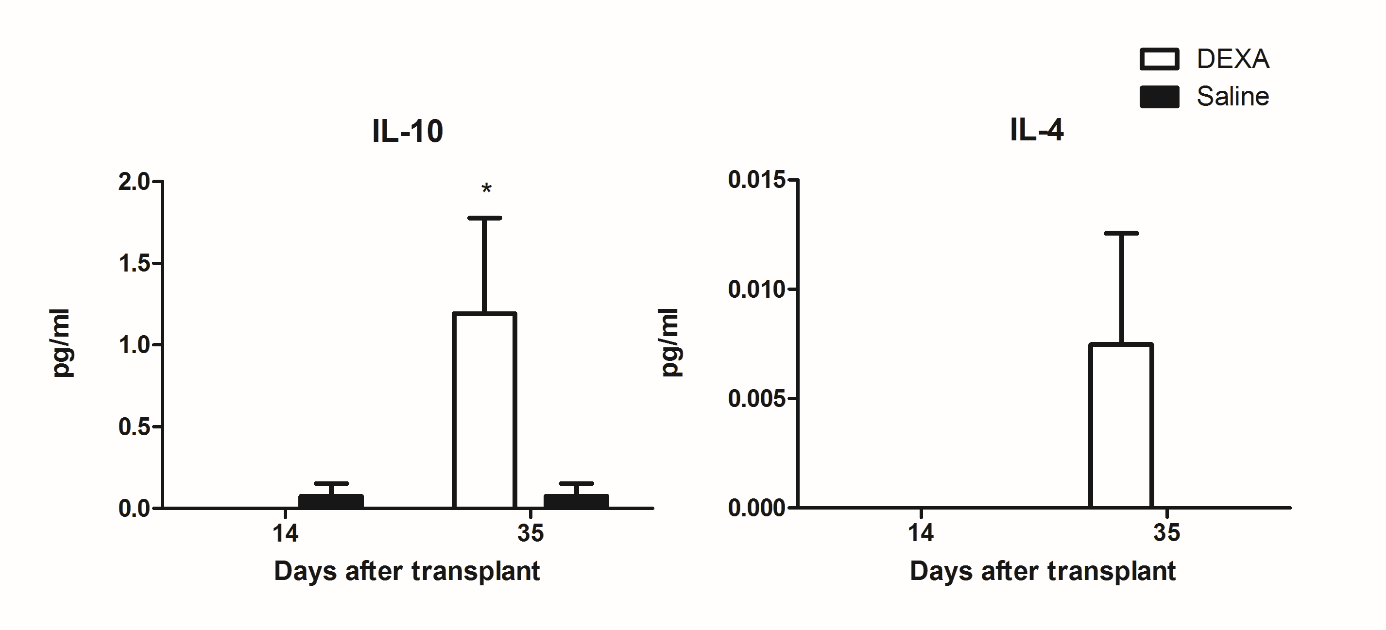

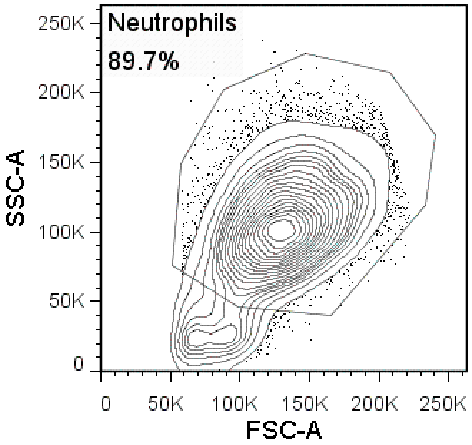

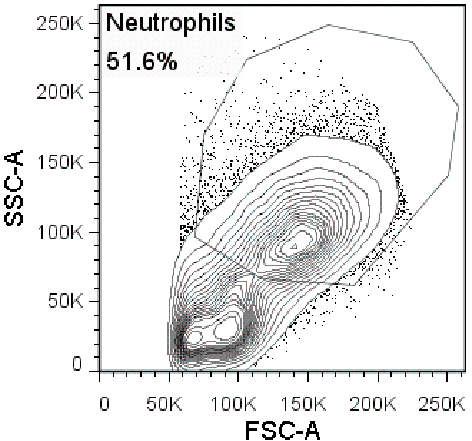


**DEXA**

**Saline**

**Figure S8. DEXA treatment induced neutrophilia in mice with autoimmune colitis.** At D28 PAT, PBLs were collected for FACS analysis. FSC^hi^SSC^hi^ neutrophils are gated in the FSC vs SSC contours. Percentages of neutrophils in PBLs from DEXA and saline control mice are compared (DEXA, n = 10; saline, n = 13) and represented by Means ± SD. (****p < 0.0001).


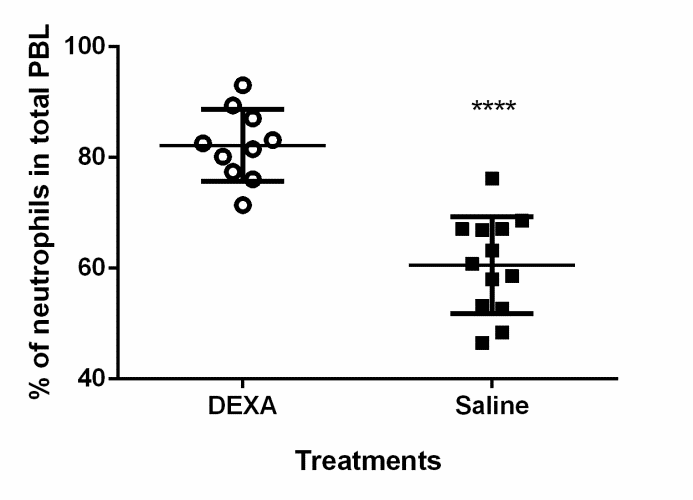


Supplemental Figure 8

Supplemental Figure 9

**Figure S9.  Production of Luc Tg. mice.** (a) Schematic representation of the construct of Luc Tg. mice. Human Ubiquitin C promoter (hUBC) is followed by cDNA of mKate fluorescent protein. Complementary DNA of mKate and luciferase are separated with a retroviral internal ribosomes entry segment (IRES). Two bovine growth hormone polyadenylation (BGHpA) signals following by two reversed direction chicken 5’-HS4 insulators (c-HS4) were ligated behind luciferase cDNA. (b) Genotyping of Luc mice. A 510-bp PCR product is amplified from genomic DNA of Luc mice.

**(a)**

**(b)**


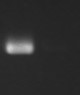


Tg

(+) (-)

510 bps


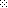

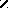

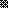

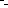


hUBC promoter

mKate

IRES

Luciferase

1 Kb


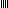

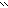


BGHpA

c-HS4
